# High-Altitude Headache Resolving After Self-Administration of Goreisan: A Case Report

**DOI:** 10.7759/cureus.114921

**Published:** 2026-08-21

**Authors:** Masahito Katsuki, Jesus Gutierrez-Arratia, Carlo Piero Lastarria, Myriam Obesso Uribe, Yasuhiko Matsumori

**Affiliations:** 1 Physical Education and Health Centre, Nagaoka University of Technology, Nagaoka, JPN; 2 Insight Research Ireland Centre for Data Analytics and School of Health and Human Performance, Dublin City University, Dublin, IRL; 3 Biostatistics, Saitama Medical University, Saitama, JPN; 4 Neurology, Instituto Nacional de Ciencias Neurológicas, Lima, PER; 5 Neurology, Asociación de cefaleas del Perú (ACEFA), Lima, PER; 6 Neurology, Sanna Clinic, Arequipa, PER; 7 Neurology, University of Piura, Lima, PER; 8 Neurology, Grau Emergency Hospital, Lima, PER; 9 Neurological Surgery, Sendai Headache and Neurology Clinic, Sendai, JPN

**Keywords:** acetazolamide therapy, acute mountain sickness, episodic migraine, goreisan (tj-17), high-altitude headache (hah), kampo medicine (japanese herbal medicine), oryeongsan/wulingsan, peru, traditional medicine, travel health

## Abstract

High-altitude headache (HAH) is the most common neurological manifestation of rapid ascent to high altitude and may occur independently or as part of acute mountain sickness (AMS). Although simple analgesics are commonly used for symptomatic treatment, evidence regarding alternative therapeutic options remains limited. Goreisan, a traditional Japanese Kampo medicine, has been reported to regulate body fluid homeostasis through aquaporin modulation and may influence cerebral fluid dynamics. However, to our knowledge, its use for HAH has not previously been reported. We herein describe a 34-year-old Japanese man with a history of migraine and hypertension, who developed a headache clinically consistent with HAH that resolved after oral administration of Goreisan. He developed a right unilateral headache approximately 90 minutes after rapid ascent from Lima (150 m) to Cusco, Peru (3,400 m). The clinical presentation was consistent with the International Classification of Headache Disorders, 3rd edition criteria for HAH, although an altitude-triggered migraine attack could not be completely excluded. Because no analgesics were available, he self-administered 2.5 g of Goreisan. The headache began to improve within 30 minutes, decreased from 4/10 to 1/10 on the Numerical Rating Scale within one hour, and resolved completely within two hours without additional medication. No recurrence occurred during the remainder of his 10-day stay, and he was able to undertake hiking activities the following day. Although improvement occurred temporally after Goreisan administration, spontaneous improvement or early acclimatization cannot be excluded, and causality cannot be established from this single case. This observation supports prospective evaluation of Goreisan in patients with HAH.

## Introduction

Rapid ascent to high altitude can induce high-altitude headache (HAH) [1] and acute mountain sickness (AMS), particularly above 2,500 m [2]. HAH is the most common neurological symptom of high-altitude exposure and may occur independently or as part of AMS [1], affecting more than 30% of climbers and travelers [3]. Previous studies have identified a history of migraine, lower arterial oxygen saturation, poor exercise tolerance, insufficient hydration, and reduced venous return as potential risk factors for HAH [3,4]. As an acute treatment, most cases respond to simple analgesics such as acetaminophen or ibuprofen [3]. As preventive treatment, acetazolamide is the standard pharmacological agent for AMS prevention because it accelerates acclimatization by enhancing ventilatory adaptation to hypobaric hypoxia [2,5]. However, acetazolamide is not universally used by travelers, and practical acute treatment for headache attacks that develop immediately after rapid ascent remains limited [5].

Kampo medicine is a Japanese traditional herbal medicine integrated into contemporary clinical practice. In Japan, Kampo medicines, including Goreisan, are described as treatment options for headache in the Clinical Practice Guideline for Headache Disorders 2021 [6]. Goreisan (TJ-17, Tsumura & Co., Tokyo, Japan), a traditional Japanese Kampo medicine, has long been prescribed for conditions associated with disturbed fluid homeostasis, including headache, dizziness, nausea, and edema [6]. Preclinical studies have suggested that Goreisan modulates cerebral blood-flow responses to barometric-pressure fluctuations [7] and may influence cerebral water transport through AQP4-related mechanisms [8,9]. In addition, Goreisan has also been reported to alleviate headaches associated with changes in weather and atmospheric pressure [10]. These findings and practice raise the possibility that Goreisan may also have a role in headaches occurring after rapid exposure to high altitude. However, evidence supporting this indication is currently lacking.

Here, we report a case of HAH that developed shortly after rapid ascent to Cusco, Peru (approximately 3,400 m) [2] and resolved promptly after oral administration of 2.5 g of Goreisan. This case describes the temporal association between the Goreisan administration and the resolution of HAH and provides a rationale for prospective evaluation of Goreisan in altitude-related headache.

This is a single case report; therefore, institutional review board approval was not required. Written informed consent was obtained from the patient for publication of this case report.

## Case presentation

A 34-year-old Japanese male presented with a headache shortly after rapid ascent to high altitude. His medical history included hypertension diagnosed at 20 years of age, which was well controlled with nifedipine 80 mg/day, carvedilol 20 mg/day, and azilsartan 40 mg/day (blood pressure approximately 120/65 mmHg). He had a history of episodic migraine without aura since the age of 14 years. His current migraine frequency was approximately one monthly headache day. He used 500 mg of paracetamol only occasionally as acute treatment. His Headache Impact Test-6 score was 40, indicating little or no headache impact [11,12], and his Migraine Interictal Burden Scale-4 score was 0, indicating no interictal burden [13,14]. He did not smoke or drink alcohol and worked as a university faculty member without shift work or excessive occupational stress. He routinely walked at least 10,000 steps every day and did strength training. His height was 179 cm, and his weight was 85 kg (body mass index 26.5 kg/m²).

After spending five days in Lima, Peru (approximately 150 m above sea level) in July (winter in Peru), he traveled by flight to Cusco (approximately 3,400 m). During his stay in Peru, he had not experienced any health issues or noticed any changes in his environment that might lead to dehydration. He departed Lima at approximately 19:00 and arrived in Cusco at 20:00. He remained asymptomatic immediately after arrival. Approximately 90 minutes after landing, he developed a persistent unilateral headache involving the right postauricular region and extending to the right temporal area. The pain was dull and pressure-like in quality and was 4/10 on the Numerical Rating Scale (NRS). The headache was aggravated by movement but was not accompanied by nausea, vomiting, dizziness, photophobia, phonophobia, or osmophobia, and the patient described it as phenotypically distinct from his habitual migraine attacks, which are typically moderate-intensity, holocranial headaches accompanied by photophobia, phonophobia, nausea, and difficulty with physical activity because of worsening of the headache with movement. In one self-assessment, he had no focal neurological symptoms and no neck stiffness. According to data recorded by a Fitbit Inspire 3 (Google LLC, Mountain View, CA, USA) wearable device, his heart rate during the headache episode was approximately 100 beats/min, and peripheral oxygen saturation was approximately 92%. Review of the device history showed that his usual oxygen saturation at low altitude was approximately 98%. During the remainder of his stay in Cusco, the recorded oxygen saturation remained around 92% without any abrupt change. These wearable-derived measurements were not independently confirmed using a medical-grade device. Blood pressure could not be measured.

Because he did not bring analgesics, he orally self-administered Goreisan extract granules (TJ-17, Tsumura & Co., Tokyo, Japan) 2.5 g, which had previously been prescribed as an acute treatment for migraine attacks and represented the prescribed single dose. Goreisan had originally been prescribed by a headache specialist approximately seven years before the present episode. Since then, the patient had used Goreisan approximately four times per month, either as an acute treatment for headache attacks or preemptively when he anticipated headache development, particularly during unfavorable weather or after insufficient sleep. He reported that Goreisan had generally been effective in both settings and that additional analgesic medication was required only approximately once per year. He had not experienced any adverse effects during previous use.

The headache began to improve approximately 30 minutes after Goreisan administration, decreased to NRS 1/10 within approximately 60 minutes, and resolved completely within two hours. No additional medication was required. No dizziness, presyncope, syncope, palpitations, or other adverse symptoms were reported after Goreisan administration. Blood pressure was not measured during or after the episode. He slept normally that night and experienced no recurrence of headache during the remainder of his stay in Cusco for a total of 10 days. Follow-up was based on patient self-report, and no subsequent in-person clinical evaluation was performed. He was able to undertake hiking activities the following day without limitation. The timeline is shown in Table 1.

**Table 1:** Clinical timeline of the high-altitude headache episode Heart rate and SpO₂ were recorded using a Fitbit Inspire 3 (Google LLC, Mountain View, CA, USA) wearable device. Follow-up during the remainder of the 10-day stay in Cusco was based on patient self-report. NRS: Numerical Rating Scale; SpO₂: peripheral oxygen saturation

Time	Clinical event
Approximately 19:00	Departure by air from Lima (approximately 150 m)
Approximately 20:00	Arrival in Cusco (approximately 3,400 m)
Approximately 21:30	Onset of right-sided pressure-like headache, NRS 4/10
During the headache episode	Heart rate approximately 100 beats/min and SpO₂ 92%
Approximately 21:40	Oral self-administration of Goreisan 2.5 g
Approximately 30 min after administration	Headache began to improve
Approximately 60 min after administration	Headache decreased to NRS 1/10
Approximately 120 min after administration	Complete headache resolution
Following day	No recurrence; hiking activities performed without limitation
Remainder of 10-day stay in Cusco	No recurrence of headache was reported based on patient self-report

According to the International Classification of Headache Disorders, 3rd edition (ICHD-3) [3], the clinical presentation was consistent with HAH because the headache developed after ascent above 2,500 m, occurred in temporal relation to the ascent, and exhibited characteristic features including mild-to-moderate intensity and aggravation by movement. However, given the unilateral location and the patient's history of migraine, an altitude-triggered migraine attack could not be completely excluded.

Although the improvement occurred temporally after the Goreisan administration, the episode was mild and self-limited, and spontaneous improvement or early acclimatization cannot be excluded. Therefore, the temporal association does not establish a causal treatment effect.

Alternative diagnoses were considered: a migraine attack triggered by altitude could not be entirely excluded given the patient's history, but the attack lacked his habitual migraine phenotype and associated migrainous features. The headache was pressure-like, lacked nausea, photophobia, phonophobia, and osmophobia, and was recognized by the patient as distinctly different from his habitual migraine phenotype; AMS was unlikely in the absence of accompanying systemic symptoms; and secondary headache disorders, including subarachnoid hemorrhage, arterial dissection, and cerebral venous thrombosis, could not be formally excluded because no independent neurological examination, blood pressure measurement, laboratory evaluation, or neuroimaging was performed. However, they were considered clinically less likely based on the reported mild and self-limited course, absence of reported focal neurological symptoms or meningism, and complete recovery without subsequent neurological symptoms. The episode was therefore classified as a HAH, while acknowledging the diagnostic overlap with migraine discussed below.

## Discussion

To our knowledge, this is the first published case describing HAH resolution following self-administration of Goreisan after rapid ascent. Although the headache improved temporally after Goreisan administration, spontaneous improvement, early acclimatization, and an expectation effect cannot be excluded. Therefore, this observation does not establish a causal treatment effect but provides a rationale for prospective evaluation of Goreisan in HAH.

Mechanism of HAH

The pathophysiology of HAH is complex and remains incompletely understood. Hypobaric hypoxia induces compensatory cerebral vasodilation to maintain oxygen delivery to the brain [4]. This adaptive response may increase cerebral blood flow and intracranial vascular distension, resulting in activation of the trigeminovascular system [4]. Subsequent release of vasoactive and inflammatory mediators, including nitric oxide, prostaglandins, calcitonin gene-related peptide, and pituitary adenylate cyclase-activating polypeptide, is thought to promote trigeminal sensitization and amplify headache [1]. In parallel, hypoxia activates hypoxia-inducible factor-1α (HIF-1α), which upregulates downstream pathways such as vascular endothelial growth factor (VEGF) [1]. Excessive activation of these pathways may increase blood-brain barrier (BBB) permeability and alter cerebral fluid homeostasis, potentially contributing to vasogenic edema and headache development [15].

In addition to hypoxia-induced neurovascular responses, disturbances in cerebral fluid homeostasis have been proposed to contribute to the development of HAH and AMS [1]. Although early hypotheses attributed HAH primarily to cerebral edema, recent evidence suggests that the increase in brain volume is generally modest and insufficient to explain headache fully [1,16,17]. Instead, hypoxia-induced activation of HIF-1α and downstream mediators such as VEGF may transiently increase BBB permeability, alter water distribution, and disturb intracranial fluid homeostasis [1]. Also, clinical observations indicate that systemic dehydration, fluid intake below 2 L during ascent, and lower arterial oxygen saturation are associated with an increased risk of HAH [1,4]. These findings suggest that dysregulated water homeostasis, rather than simply dehydration or cerebral edema alone, may contribute to altitude-related headache.

Taken together, although the precise pathophysiology of HAH remains incompletely understood, current evidence suggests that hypobaric hypoxia initiates a cascade of neurovascular, inflammatory, and fluid-regulatory responses. This concept provides a biological rationale for considering therapies that modulate intracranial fluid regulation in addition to conventional approaches targeting hypoxic adaptation.

Possible pharmacological mechanism of Goreisan

Goreisan is a traditional Japanese Kampo medicine that has long been used to treat disorders associated with impaired body fluid homeostasis, including edema, dehydration, vertigo, nausea, and weather-related headache. Unlike conventional diuretics, Goreisan has traditionally been regarded as less likely to cause excessive dehydration in clinical practice [18]. Experimental studies suggest that Goreisan can modulate aquaporin-4 (AQP4)-mediated cerebral water transport. In a murine ischemic stroke model, repeated Goreisan administration reduced brain water content and suppressed pathological AQP4 upregulation [8]. In experimental systems expressing AQP4, Goreisan has also been reported to inhibit AQP4-mediated water permeability, supporting a potential direct effect on AQP4 function [9]. These preclinical findings suggest that Goreisan may influence pathological cerebral water regulation rather than acting solely as a simple diuretic.

**Figure 1:**
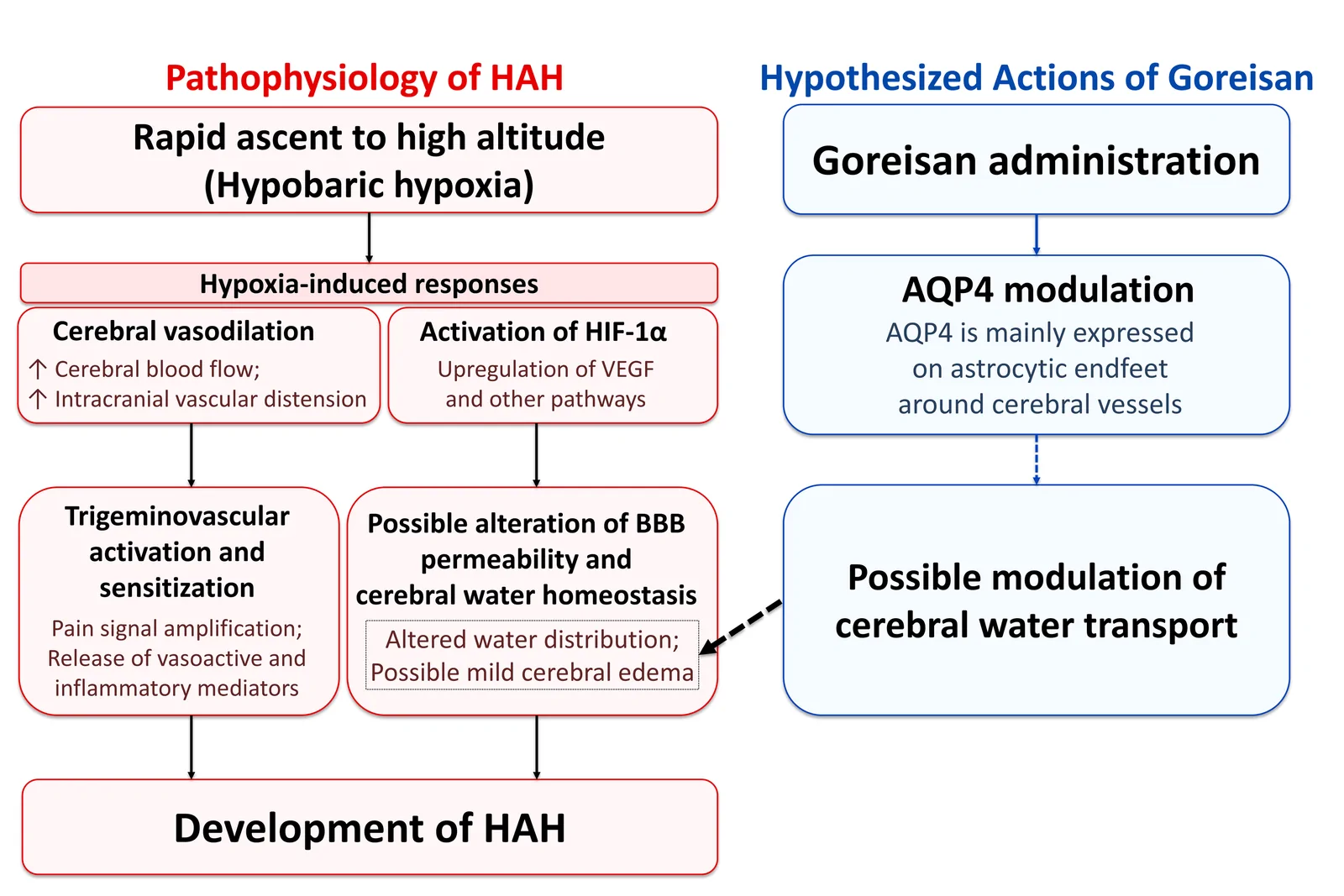
Proposed mechanisms of high-altitude headache (HAH) and hypothesized actions of Goreisan Rapid ascent causes hypobaric hypoxia, which may promote cerebral vasodilation, trigeminovascular activation, and hypoxia-inducible factor-1α (HIF-1α)/vascular endothelial growth factor (VEGF)-related alterations in blood–brain barrier (BBB) permeability and cerebral water homeostasis. Experimental studies suggest that Goreisan can modulate aquaporin-4 (AQP4)-mediated cerebral water transport; however, whether this mechanism contributes to symptom relief in HAH remains unknown. Dashed arrows indicate hypothesized actions of Goreisan that have not been demonstrated in HAH. No mechanistic measurements were obtained in the present patient; therefore, the Goreisan-related pathway shown in this figure represents a conceptual hypothesis rather than case-specific mechanistic findings.

Although modulation of cerebral water homeostasis represents a possible biological hypothesis, its relevance to symptom resolution in the present case remains unknown. Hypobaric hypoxia may alter BBB permeability and cerebral water distribution, while preclinical studies have shown that Goreisan can affect AQP4 expression and AQP4-mediated water permeability (Figure 1) [8,9].

However, these findings have not been demonstrated in HAH, and no measurements of cerebral water dynamics, BBB permeability, or AQP4 activity were obtained in the present patient. Therefore, the AQP4-related pathway shown in Figure 1 should be regarded only as a hypothesis for future investigation rather than an explanation for the clinical improvement observed in this case.

From a practical standpoint, most cases of HAH respond to simple analgesics such as acetaminophen or ibuprofen, while acetazolamide is used for AMS prophylaxis [1]. However, travelers frequently ascend without carrying these agents, as occurred in the present case. Unlike conventional loop diuretics, Goreisan has traditionally been regarded as less likely to cause excessive dehydration in clinical practice [18], a potentially relevant property at high altitude, where dehydration itself aggravates HAH. These features, together with its reported clinical use for primary headache in Japan [19], make Goreisan a plausible candidate for prospective evaluation in altitude-related headache, particularly for individuals who already use it for primary headache. Importantly, this observation should not be interpreted as supporting Goreisan as a substitute for established measures for altitude illness, including appropriate acclimatization, adequate hydration, descent when clinically indicated, oxygen therapy when required, and medical evaluation of concerning symptoms.

Limitations and considerations

This report has several limitations. First, this was a single, uncontrolled case, and spontaneous improvement, early acclimatization, or the natural course of HAH cannot be excluded. Second, an expectation effect is possible because the patient had previously used Goreisan as an acute treatment for migraine and self-administered it with an expectation of benefit. Third, oxygen saturation and heart rate were recorded using a consumer wearable device rather than a validated medical-grade monitoring device, and blood pressure was not measured during the episode. Fourth, no independent neurological examination, laboratory evaluation, or neuroimaging was performed; therefore, secondary causes of headache could not be formally excluded. Fifth, because the patient had a history of episodic migraine and the headache was unilateral, an altitude-triggered migraine attack could not be completely excluded, although the episode lacked his habitual migrainous features and was considered clinically consistent with HAH. Finally, no mechanistic measurements related to cerebral water dynamics or AQP4 activity were obtained; therefore, the proposed pharmacological mechanism of Goreisan remains speculative.

## Conclusions

We report a case of HAH that resolved following self-administration of Goreisan after rapid ascent to approximately 3,400 m. Although improvement occurred temporally after Goreisan administration, causality cannot be established from this single case because spontaneous improvement, early acclimatization, and an expectation effect cannot be excluded. This observation supports prospective evaluation of Goreisan in patients with HAH.
